# Association between the baseline gene expression profile in periapical granuloma and periapical wound healing after surgical endodontic treatment

**DOI:** 10.1038/s41598-022-17774-z

**Published:** 2022-08-15

**Authors:** Muhammad Adeel Ahmed, Fizza Nazim, Khalid Ahmed, Muhammad Furqan Bari, Abdulaziz Abdulwahed, Ahmed A. AlMokhatieb, Yaseen Alalvi, Tariq Abduljabbar, Nouman Mughal, Syed Hani Abidi

**Affiliations:** 1 Department of Restorative Dental Sciences, College of Dentistry, King Faisal University, Al-Ahsa, Saudi Arabia; 2 Department of Biological and Biomedical Sciences, Aga Khan University, Karachi, Pakistan; 3 Department of Pathology, Dr. Ishrat-ul-Ebad Khan Institute of Oral Health Sciences, Dow University of Health Sciences, Karachi, Pakistan; 4 Department of Conservative Dental Sciences, College of Dentistry, Prince Sattam Bin Abdulaziz University, Al-Kharj, Saudi Arabia; 5 College of Dentistry, King Saud University, Riyadh, Saudi Arabia; 6 Department of Prosthetic Dental Science, College of Dentistry, King Saud University, Riyadh, Saudi Arabia; 7 Department of Surgery, Aga Khan University, Karachi, Pakistan; 8 Department of Biomedical Sciences, Nazarbayev University School of Medicine, Nur-Sultan, Kazakhstan

**Keywords:** Molecular biology, Biomarkers

## Abstract

In this study, we have investigated the association between the baseline gene expression profile in periapical granuloma and periapical wound healing after surgical endodontic treatment. Twenty-seven patients aged between 15 and 57 years underwent periapical surgery. The retrieved periapical tissue sample was used for mRNA expression analysis of *COL1A1, VTN*, *ITGA5, IL-4, TNF*, *ANGPT, VEGFA*, and *CTGF*. All patients were recalled after 6 and 12 months for periapical healing evaluation. Healing was then correlated with baseline gene expression. Healing was observed in 15 patients at the end of 6 months, which increased to 21 patients after 12 months. Six patients showed no healing even after 12 months. Analysis of baseline expression levels of the tested genes with healing status showed the mean relative expression of *VTN, VEGFA, ANGPT, TNF, and CTGF* to be significantly different (*p* < 0.05) between the healing group (6 and 12 months) (72.99%) and the non-healing (94.42%) group. Periapical Index scores 3–5 exhibited a positive correlation with *ITGA-5* expression. Overexpression of *ANGPT* and a strong positive correlation between *ITGA5* and PAI scores in the non-healing group of patients may suggest these genes to be a potential prognostic biomarker for periapical wound non-healing after surgical endodontic treatment.

## Introduction

Periapical periodontium can be damaged by periapical lesions associated with inflammatory processes^1^. The chronic form of such condition is known as periapical granuloma (PG), which involves scarring and the formation of granulation tissue with concomitant infiltration of chronic inflammatory cells, such as macrophages, mast cells, lymphocytes, and plasma cells^2^. The inflammation in the periapical tissues, often followed by bacterial infection, is mediated by immunoregulatory mediators, chemokines, and cytokines of proinflammation which degrade the extracellular matrix (ECM) and cause the periapical bony erosion^3^.

Wound healing in periapical granuloma involves the interaction of the cells and the extracellular matrix. It involves the activation of genes that are associated with the formation of extracellular components, enzymes that remodel the microenvironment, expression of cell adhesion molecules, and expression of chemokines, cytokines, and growth factors^4^. Inflammatory cytokines that are required for healthy wound healing may also destroy the healing tissue in certain conditions^5^. This change of effect from healing to destruction can be explained by the duration and nature of the host immune response involving intricate cell signaling pathways^6^.

Following surgical endodontic treatment of periapical lesions, the healing processes do not remain consistent across patients, where different patients respond differently to the treatment^7^. The differences in the genetic polymorphisms of the individual patients, and the corresponding change in the expression of different genes, which are involved in wound healing are responsible for such a change^8^. For example, matrix metalloproteinases (MMP) play a significant role in bone healing^9^, and a high concentration of MMP-9 has been found in patients with periapical granuloma suggesting that it has a role in wound remodeling. Similarly, in chronic apical abscess, the higher expression levels of MMP-9 and MMP-7 have been reported and shown to be associated with increased levels of destruction in the tissue by changing dynamics of inflammation in the periapical lesion^10^. Similarly, we have recently shown overexpression of MMP2 and MMP9 to be associated with the outcome of periapical wound healing after surgical endodontic treatment^11^.

In addition to MMPs, differential expression of certain other genes which are involved in extracellular matrix formation *(COL1A1, VTN),* cell adhesion (*ITGA5*), inflammatory cytokines & chemokines (*IL-4, TNF*), growth factors (*ANGPT1, VEGFA*), and signal transduction pathways (*CTGF*), etc. have been associated with different phases of wound healing in periapical lesions^12^. For example, up-regulation of *COL1A1, VTN, TNF, CTGF*, and *ITGA5* has been reported in healing periapical lesions as compared with adjacent healthy periapical tissue^12^.

To the best of our knowledge, no study has reported the baseline expression of wound healing marker genes, viz. *COL1A1, VTN, ITGA5, IL-4, TNF, ANGPT1, VEGFA,* and *CTGF* in periapical granuloma after surgical endodontic treatment, and its subsequent correlation with healing/non-healing as an outcome parameter.

## Methods

### Patient selection

Study participants were selected from a pool of patients referred to the Department of Operative Dentistry, Dow University of Health Sciences from November 2017 to October 2019. The study protocol was approved by Institutional Review Board, Dow University of Health Sciences (Ref: IRB-862/DUHS/Approval/2017/50). The samples were collected after obtaining written informed consent from all participants. In the case of minors, informed consent from a parent and/or legal guardian of the subject was obtained. All methods were performed as per the relevant guidelines and regulations.

Participants aged between 15 and 57 years, who presented with chronic apical periodontitis or chronic apical abscess of an anterior tooth with previously attempted or failed root canal treatment were enrolled in this study. Initially, a total of 52 patients who met the inclusion criteria of the study were recruited in the study. First, conventional re-root canal treatment was performed, and these patients were recalled for follow-up for up to 6 months and periapical healing was evaluated both clinically and radiographically. Those patients (n = 27) in which healing was not evident after conventional re-root canal treatment underwent periapical surgery. Exclusion criteria were medically compromised patients with any uncontrolled systemic disease or ASA Level III, multi-rooted teeth, single-rooted teeth with less than 4 mm periapical lesion, histopathology evaluation showed the presence of cyst rather than periapical granuloma and patients in which the healing was evident after conventional re-root canal treatment.

### Treatment protocol and tissue retrieval

Before initiating periapical surgery, a preoperative digital periapical radiograph was taken for all patients as a baseline using paralleling technique, cone indicator, and a radiopaque reference marker placed over the sensor to ensure the constant distance and angle between the x-ray cone and sensor on every shoot. Periapical surgery was performed using protocols as described previously^11^. Briefly, the full-thickness mucoperiosteal flap was raised and the periapical lesion site was identified. Access to the lesion was gained through a window preparation. After removal of the periapical lesion, a retrograde cavity was prepared by ultrasonic tip (Pro ultra, DENTSPLY Maillefer, Switzerland), followed by retrograde filling with MTA (Pro-root MTA, DENTSPLY Tulsa Dental Specialties, USA). Retrieved periapical tissue was stored for histopathological and gene expression analysis. All patients were recalled after 6 and 12 months for the evaluation of periapical wound healing based on clinical and radiographic healing criteria. The clinical criteria for healing were the absence of swelling/pain/sinus tract and/or tenderness to percussion, while the radiographic healing was assessed using the periapical index (PAI). PAI scores 1 and 2 were regarded as healing, whereas PAI scores 3–5 were considered non-healing. Three different examiners independently evaluated the radiographs and the specific healing score which two examiners agreed was accepted.

### Tissue processing for RNA extraction and cDNA synthesis

Frozen periapical granuloma tissue samples were used to purify total RNA, according to the instructions of the manufacturer with the help of a bead mill homogenizer. Briefly, each tissue was homogenized using Omni bead ruptor 24 (Omni International, Kennesaw, GA, USA) in presence of RLT Buffer (500µL containing 1% β-mercaptoethanol). RNeasy Mini kit (Qiagen, Hilden, Germany) was used to purify total RNA from the homogenate using the manufacturer’s instructions. The extracted RNA was stored at − 80 °C till further use.

RNA was reverse transcribed by using an M-MLV reverse transcriptase kit (Promega, Madison, WI, USA). RNA template (5 µL) was mixed with 1µL OligodTs (0.5 µg/µL), 1 µL dNTPs 10 µM, and 8 µL Nuclease free water and was incubated on the preheated block at 65 °C for 5 min. At the end of incubation, the reaction mixture was immediately chilled on ice for 5 min then briefly spin to bring the contents down to the bottom of the tube. This reaction was combined with a reaction mixture containing 4 µL M-MLV RT 5 × reaction buffer and 1 µL M-MLV Reverse transcriptase (10,000U) to make the volume up to 20 µL. This reaction mixture was incubated at 50 °C for 30 min, 85 °C for 5 min, and 4 °C for hold in Eppendorf (Hamburg, Germany) thermal cycler.

### Quantitative polymerase chain reaction for *COL1A1, VTN*,* ITGA5, IL-4, TNF*, *ANGPT, VEGFA*, and *CTGF* genes

cDNA samples were used to perform quantitative-PCR (qPCR) to measure the expression levels of COL1A1, VTN, ITGA5, IL-4, TNF, ANGPT, VEGFA, and *CTGF* genes. β-actin and β-globin served as housekeeping genes and the average of both housekeeping genes was also used to normalize the results in a qPCR using respective primer sets. A list of primers used to measure the levels of *COL1A1, VTN*, *ITGA5, IL-4, TNF*, *ANGPT, VEGFA*, *CTGF*, and β-actin is given in Table 1.

**Table 1:** Name of target genes and respective primer sets used to quantify mRNA levels in qPCR.

Gene	Forward primer (5′–3′)	Reverse primer (5′–3′)
*β-actin*	GCGCGGCTACAGCTTCA	CTCCTTAATGTCACGCACGAT
*β-globin*	ACACAACTGTGTTCACTAGC	CAACTTCATCCACGTTCACC
*COL1A1*	GAGGGCCAAGACGAAGACATC	CAGATCACGTCATCGCACAAC
*VTN*	TGACCAAGAGTCATGCAAGGG	ACTCAGCCGTATAGTCTGTGC
*ITAG-5*	GGCTTCAACTTAGACGCGGAG	TGGCTGGTATTAGCCTTGGGT
*IL-4*	CCAACTGCTTCCCCCTCTG	TCTGTTACGGTCAACTCGGTG
*TNF*	GAGGCCAAGCCCTGGTATG	CGGGCCGATTGATCTCAGC
*ANGPT1*	AGCGCCGAAGTCCAGAAAAC	TACTCTCACGACAGTTGCCAT
*VEGFA*	AGGGCAGAATCATCACGAAGT	AGGGTCTCGATTGGATGGCA
*CTGF*	CAGCATGGACGTTCGTCTG	AACCACGGTTTGGTCCTTGG

For the analysis of qPCR, 10 µL of the reaction mixture was prepared by adding: 01 µL cDNA, 0.25 µL (10 pmol/µL) reverse and forward primers, 05 µL of BrightGreen 2X qPCR MasterMix-No Dye (ABM, Canada), and nuclease-free water was added to make up the volume. CFX96™ Real-Time PCR System (BIO-RAD, USA), was used to perform the q-PCR reaction with the given protocol: 10 min at 95 °C, 40 cycles of 15 s at 95 °C, and 30 s at 60 °C. Melt curve (55 °C-95 °C) analysis was performed at the end of 40/60 cycles to verify the identity of PCR products. All reactions were run in duplicate. The relative gene expression was calculated using the comparative Ct (threshold cycle) method^13,14^.

### Statistical analysis

We applied the Unpaired T-test with Welch’s correction to determine the significant difference in relative gene expression between healing (6 and 12 months) and non-healing groups, as well as between periapical abscess and periapical periodontitis. Similarly, we applied the Pearson correlation test to determine the correlation between relative gene expression of different genes in the healing and non-healing group. In all analyses, a *p* < 0.05 was considered to be statistically significant. The statistical analyses were performed on IBM SPSS Statistics v.20.

## Results

A total of 27 patients, 20 males, and seven females with a mean age of 22.8 + 7.5, receiving periapical surgery were included in this study (Table 2). The PAI (Periapical Index) score of either 4 or greater than 4 was observed in all patients on preoperative periapical radiographs (Table 2).

**Table 2:** The demographic information and clinical details about the patients in the study.

Healing status	Sample ID	Age (Years)	Sex	Tooth in question	Type of lesion	Etiology of the lesion	Diagnosis	Treatment received	Size of lesion at baseline	PAI score	Size of lesion at 6 months (mm)	PAI score at 6 months	Size of lesion at 12 months (mm)	PAI Score at 12 months
					(Granuloma or abscess)					At the baseline				
Healing group at 12 months	1	19	Female	21	Abscess	FERT*	CPA**	SET****	6 mm	5	3	4	< 1	2
	2	25	Male	21	Granuloma	FERT*	CPP***	SET****	5	4	2	3	< 1	2
	3	32	Male	21 and 22	Abscess	FERT*	CPA**	SET****	7 mm	5	2	3	< 1	2
	7	15	Female	31	Abscess	FERT*	CPA**	SET****	5 mm	5	2	3	< 1	2
	8	17	Male	42	Abscess	FERT*	CPA**	SET****	5 mm	5	3	3	< 1	2
	27	15	Male	21	Granuloma	FERT*	CPP***	SET****	5 mm	4	2	4	< 1	2
Healing group at 6 months	5	20	Male	22	Granuloma	FERT*	CPP***	SET****	4 mm	4	< 1	2	< 1	2
	6	18	Male	21 and 22	Abscess	FERT*	CPA**	SET****	7 mm	5	< 1	2	< 1	2
	9	22	Female	12	Granuloma	FERT*	CPP***	SET****	4 mm	4	< 1	2	0	1
	10	15	Male	12	Granuloma	FERT*	CPP***	SET****	5 mm	4	0	1	0	1
	11	19	Male	22	Granuloma	FERT*	CPP***	SET****	4 mm	4	< 1	2	0	1
	12	17	Male	11	Abscess	FERT*	CPA**	SET****	5 mm	5	< 1	2	< 1	2
	13	26	Male	21	Granuloma	FERT*	CPP***	SET****	4 mm	4	< 1	2	0	1
	16	28	Male	31	Abscess	FERT*	CPA**	SET****	5 mm	5	< 1	2	< 1	2
	17	33	Male	12	Granuloma	FERT*	CPP***	SET****	4 mm	4	0	1	0	1
	18	19	Female	41	Granuloma	FERT*	CPP***	SET****	4 mm	4	< 1	2	< 1	2
	19	27	Female	22	Abscess	FERT*	CPA**	SET****	5 mm	5	< 1	2	< 1	2
	20	18	Female	11 and 21	Abscess	FERT*	CPA**	SET****	4 mm in 11 4 mm in 21	5	< 1	2	0	1
	24	23	Male	22	Granuloma	FERT*	CPP***	SET****	4 mm	4	< 1	2	< 1	2
	26	15	Male	11	Granuloma	FERT*	CPP***	SET****	4 mm	4	0	1	0	1
	30	18	Male	22	Granuloma	FERT*	CPP***	SET****	4 mm	4	< 1	2	0	1
No-healing	4	42	Male	22	Abscess	FERT*	CPA**	SET****	5 mm	5	3	4	1.5	3
	14	32	Female	31 and 41	Abscess	FERT*	CPA**	SET****	6 mm	5	3	4	2	3
	15	39	Male	12	Abscess	FERT*	CPA**	SET****	7 mm	5	4	4	2	3
	22	24	Male	22	Granuloma	FERT*	CPP***	SET****	5 mm	4	2	4	1	3
	23	16	Male	31 and 32	Abscess	FERT*	CPA**	SET****	8 mm	5	5	5	4	4
	29	22	Male	21	Abscess	FERT*	CPA**	SET****	6 mm	4	3	4	2	3

*FET failed endodontic treatment, **CPA chronic periapical abscess, ***CPP chronic periapical periodontitis; ****SET surgical endodontic treatment.

**Figure 1:**
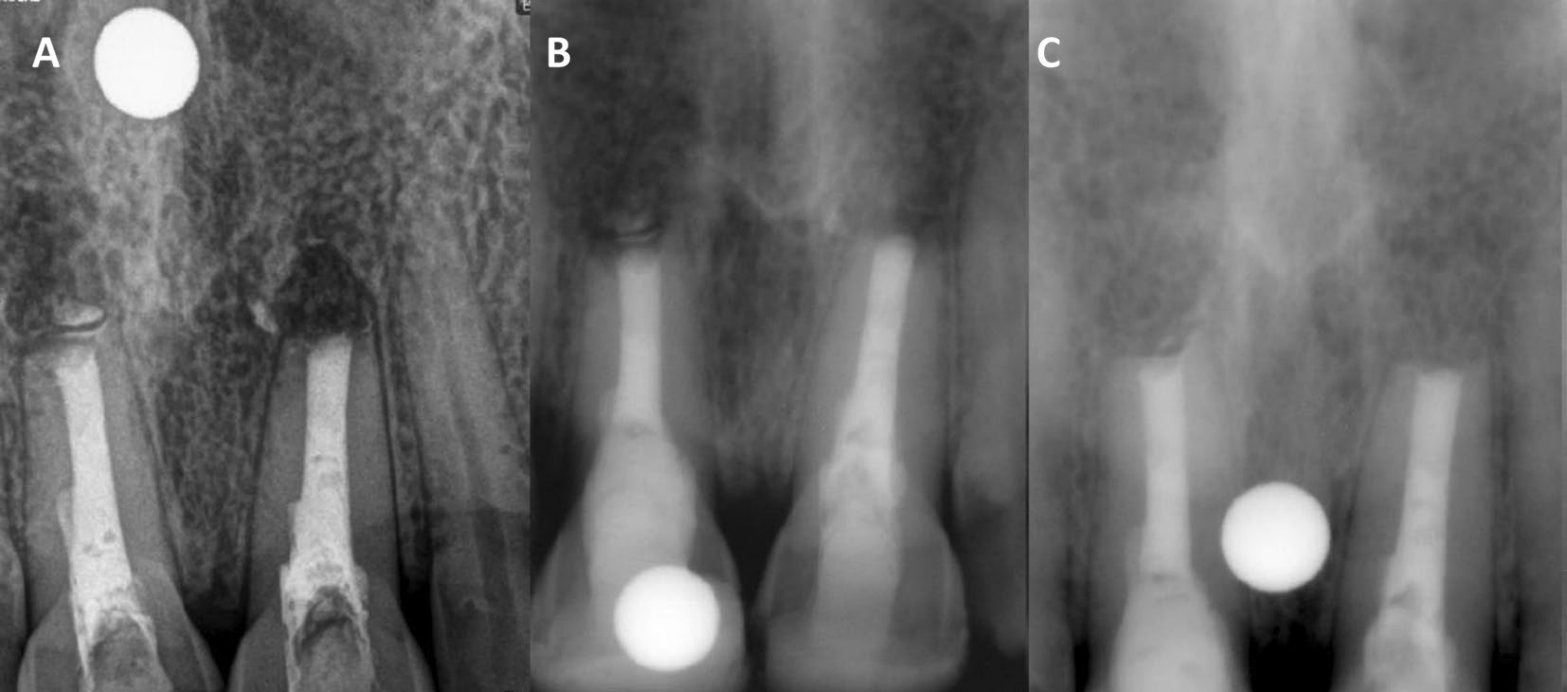
**(A)** Immediate postoperative periapical radiograph showing periapical radiolucency around the apex of Tooth 11 and 21. (**B**) Periapical radiolucency decreased in 6 months. (**C**) Complete resolution of periapical radiolucency in 1 year.

**Figure 2:**
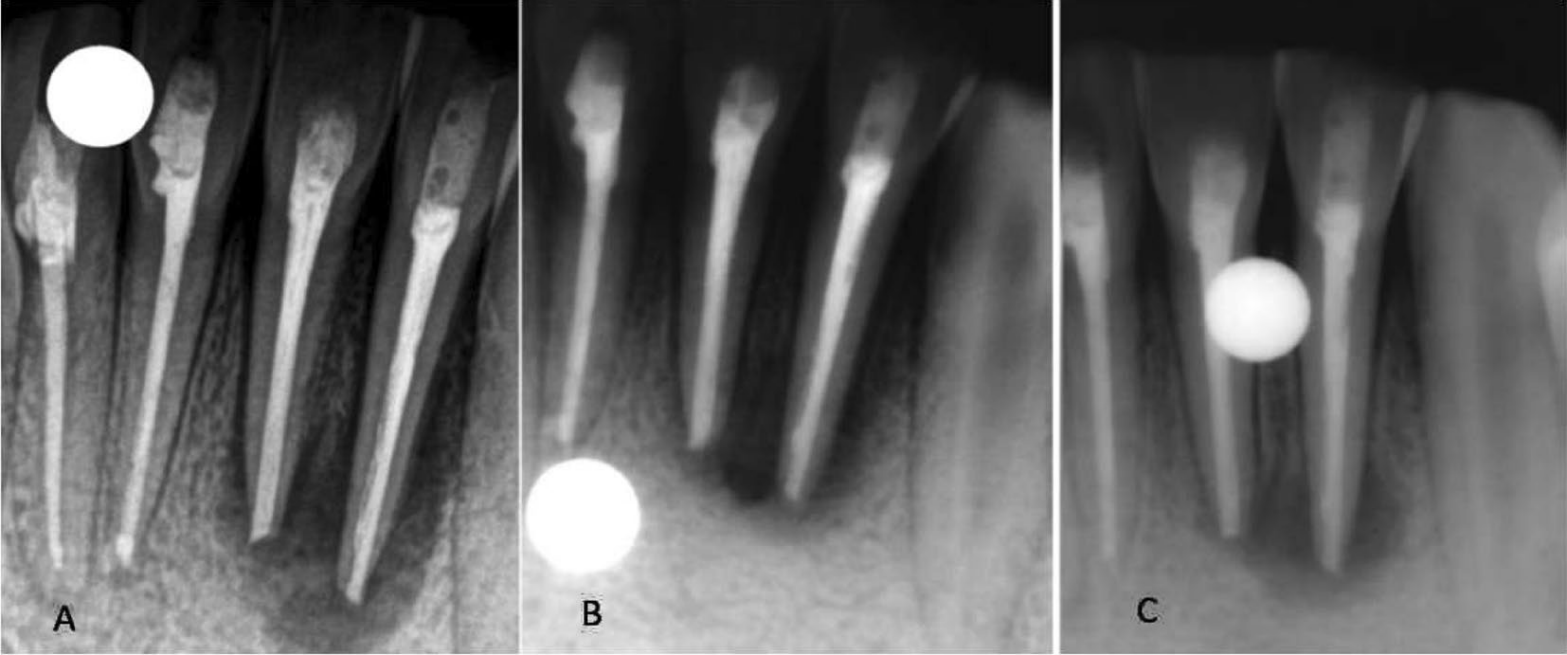
(**A**) Immediate postoperative periapical radiograph showing periapical radiolucency around the apex of Tooth 31 and 32. (**B**) Periapical radiolucency slightly decreased in 6 months. (**C**) No resolution of periapical radiolucency in 1 year.

The patients were called for follow-up after 6 and 12 months of periapical surgery for the evaluation of their healing status both radiographically and clinically. The patients who have no complaints of swelling, pain, sinus tract, and/or tenderness to percussion at the end of the follow-up period were included in the healing group. Whereas, all those patients at the follow-up who had presented with the above-mentioned signs and symptoms were included in the non-healing group. Healing and non-healing were also characterized based on radiographic presentation. Periapical Index (PAI) score was used to assess the outcome of radiographic healing (Figs. 1 and 2). PAI scores 1 and 2 were regarded as healing, whereas PAI scores 3 to 5 were considered non-healing. The radiological findings after the follow-up period are summarized in Table 2. The periapical radiolucency did not increase in any patient after 6 and 12 months. Out of 27, healing was observed in 15 (55.55%) patients after 6 months and 21 (77.77%) patients after 12 months. Twelve patients (44.44%) presented with no healing at the end of 6 months and only 6 patients (22.22%) reported no healing at the end of 12 months.

### Analysis of baseline differential expression of ***COL1A1, VTN***,*** ITGA5, IL-4, TNF***, ***ANGPT, VEGFA***, and ***CTGF*** genes in healing versus non-healing group

**Figure 3:**
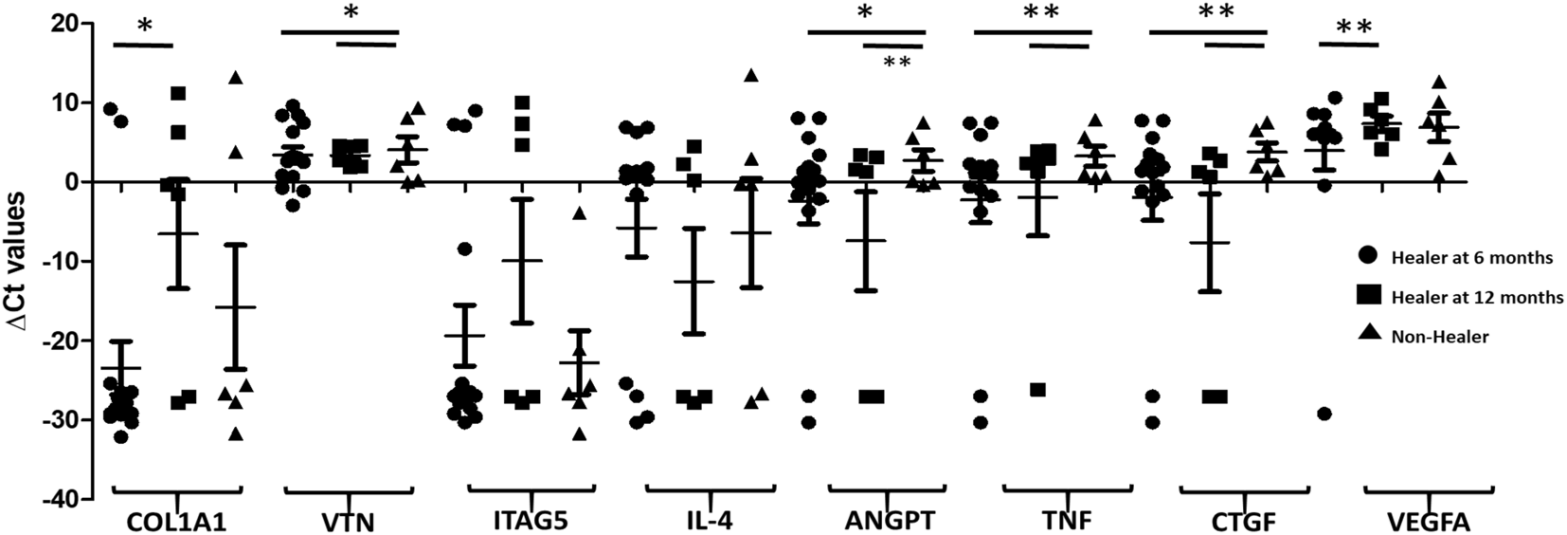
Baseline expression of *COL1A1, VTN*, *ITGA5, IL-4, TNF*, *ANGPT, VEGFA*, and *CTGF* in healing at 6- and 12-months versus non-healing group: The baseline expression of *COL1A1, VTN, ITGA5, IL-4, TNF, ANGPT, VEGFA, and CTGF* was measured in the healing at 6 months (circles), healing at 12 months (squares) and non-healing (triangles) groups. The Y-axis shows the relative expression (ΔCt) of each gene tested, symbols (circles, squares and triangles) represent each data point, and the error bars show the standard error of the mean. The lines with the asterisk sign show a significant difference (**p < 0.001, *p < 0.05) in the expression of tested genes between the healing (6–12 months) and non-healing groups.

In this study, the relative gene expression of genes associated with the extracellular matrix formation (*COL1A1, VTN)*, cell adhesion (*ITGA5*)*,* inflammatory cytokines & chemokines (*IL-4, TNF),* Growth factors (*ANGPT, VEGFA),* and signal transduction pathways (*CTGF)* in both healing (6 months and 12 months) and non-healing groups have been determined. We found that the expression of *ANGPT, TNF, and CTGF* to be significantly down-regulated in the healing group (at 6 and 12 months, viz. with healing at 6 months: − 2.38, − 2.25, − 1.92 versus with healing at 12 months: − 7.45, − 1.95, − 7.64), while the same genes were upregulated in the non-healing group (2.7, 3.26 and 3.78, respectively). Although, *COL1A1* expression was down-regulated in all three groups (Healing at 6 months, healing at 12 months, and nonhealing groups), however, a significant difference was observed between healing at 6 months and healing at 12 months (healing at 6 months: − 23.44, healing at 12 months: − 6.54; *p* < *0.01*) (Fig. 3). Similarly, the relative expression of *VEGFA* was found to be significantly different (*p* < 0.005) between the healing group at 6 months (3.97) and the healing group at 12 months (7.35).

**Figure 4:**
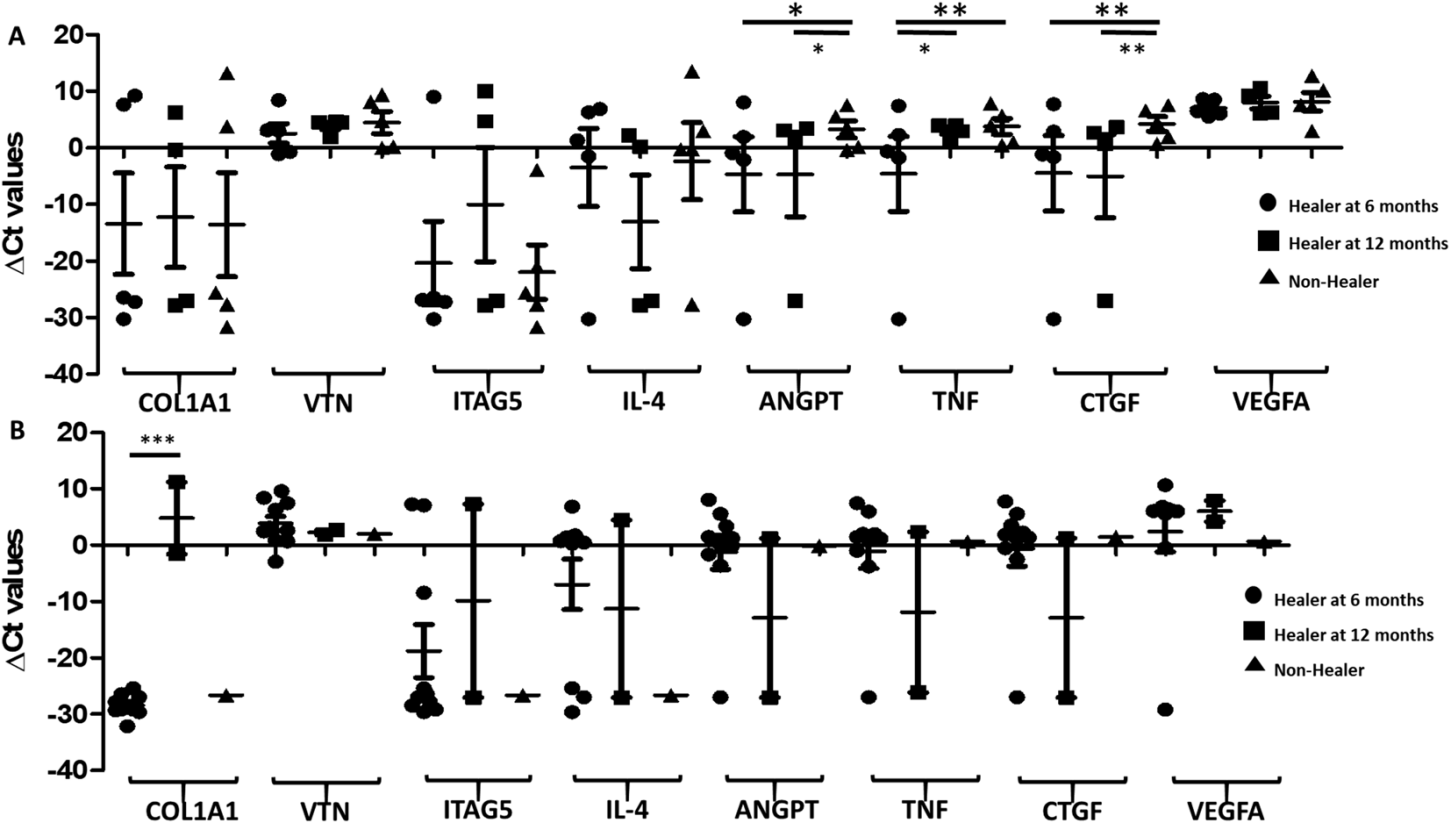
Baseline expression of *COL1A1, VTN*, *ITGA5, IL-4, TNF*, *ANGPT, VEGFA*, and *CTGF* in (**A**) periapical abscess, and (**B**) periapical periodontitis groups in healing at 6- and 12-months versus non-healing group: The baseline expression of *COL1A1, VTN, ITGA5, IL-4, TNF, ANGPT, VEGFA, and CTGF* was measured for (**A**) periapical abscess, and (**B**) periapical periodontitis lesions at 6 months (circles), 12 months (squares) and non-healing (triangles) groups. The Y-axis shows the relative expression (ΔCt) of each gene tested, symbols (circles, squares and triangles) represent each data point, and the error bars show the standard error of the mean. The lines with the asterisk sign show a significant difference (***p < 0.0001, **p < 0.001, *p < 0.05) in the expression of tested genes between the healing (6–12 months) and non-healing groups.

We have also categorized the data based on the nature of the periapical lesions (abscess and periodontitis) and further divided the group into healing at 6, 12 months, and non-healing groups (Fig. 4). In the periapical abscess group, we found the expression of *ANGPT and CTGF* to be significantly down-regulated in the healing group (*ANGPT*: healing at 6 months: − 4.66, healing at 12 months: − 4.73; *CTGF*: healing at 6 months: − 4.49, healing at 12 months: − 5.02), while upregulated in the non-healing group (*ANGPT: 3.26* and *CTGF: 4.23*; Fig. 4). A statistically significant difference was observed in the expression of *TNF* in healing at 6 months (down-regulated; − 4.58; *p* < 0.001) while up-regulated in healing at 12 months (3.02; *p* < 0.019) and non-healing group (3.78; *p* < 0.001). In the periapical periodontitis group, *COL1A1* was significantly down-regulated in healing at 6 months (− 28.44) while up-regulated in healing at 12 months group (4.83; *p* < 0.0001; Fig. 4).

In the next step, we applied the Pearson correlation test to determine the correlation in the relative expression of the *COL1A1, VTN, ITGA5, IL-4, TNF, ANGPT, VEGFA, and CTGF* genes in the healing versus non-healing group (Tables 3 and 4). In the healing group, the expression of *COL1A1* and *ITGA5; IL-4 and ITGA5, ANGPT, CTGF; ANGPT* and *TNF, CTGF;* and *TNF* and *CTGF* was found to be positively correlated (*p* < 0.05; Table 3). Similarly, in the non-healing group, the expression of *VTN* and *ANGPT*; *IL4* and *VEGFA*; *TNF* and *ANGPT, CTGF; CTGF* and *ANGPT*, *TNF;* was found to be positively correlated, whereas the expression of *VTN* and *ITGA5* was found to be negatively correlated in the non-healing group respectively (*p* < 0.05; Table 4).

**Table 3:** Correlation between relative gene expression of *COL1A1, VTN, ITGA5, IL-4, TNF, ANGPT, VEGFA, and CTGF* in the healing group.

	COL1A1	VTN	ITGA5	IL4	ANGPT	TNF	CTGF	VEGFA
*COL1A1*	–	**0.01**	0.51	0.27	− 0.12	0.11	**0.05**	0.08
*VTN*	**0.01**	–	0.08	**0.05**	0.20	0.18	0.19	0.28
*ITGA5*	0.51	0.08	–	0.52	0.19	0.12	0.38	0.06
*IL4*	0.27	**0.05**	0.52	–	0.57	0.32	0.67	− 0.13
*ANGPT*	− 0.12	0.20	0.19	0.57	–	0.49	0.89	− 0.14
*TNF*	0.11	0.18	0.12	0.32	0.49	–	0.57	**0.03**
*CTGF*	**0.05**	0.19	0.38	0.67	0.89	0.57	–	− 0.06
*VEGFA*	0.08	0.28	0.06	− 0.13	− 0.14	**0.03**	− 0.06	–

The table shows the correlation coefficient (r) value between each gene pair, where gene pairs exhibiting statistically significant (*p* < 0.05) correlation are bold.

**Table 4:** Correlation between relative gene expression of *COL1A1, VTN, ITGA5, IL-4, TNF, ANGPT, VEGFA, and CTGF* in the non-healing group.

	COL1A1	VTN	ITGA5	IL4	ANGPT	TNF	CTGF	VEGFA
*COL1A1*	–	− 0.20	0.69	0.61	− 0.26	− 0.35	− **0.02**	0.65
*VTN*	− 0.20	–	− 0.76	− **0.04**	0.74	0.66	0.48	− 0.11
*ITGA5*	0.69	− 0.76	–	0.37	− 0.44	− 0.41	− 0.09	0.44
*IL4*	0.61	− **0.04**	0.37	–	0.21	0.17	0.46	0.97
*ANGPT*	− 0.26	0.74	− 0.44	0.21	–	0.97	0.87	0.17
*TNF*	− 0.35	0.66	− 0.41	0.17	0.97	–	0.92	0.09
*CTGF*	− **0.02**	0.48	− 0.09	0.46	0.87	0.92	–	0.35
*VEGFA*	0.65	− 0.11	0.44	0.97	0.17	0.09	0.35	–

The table shows the correlation coefficient (r) value between each gene pair, where gene pairs exhibiting statistically significant (*p* < 0.05) correlation are bold.

### Correlation between PAI score based on the radiologic assessment and relative expression of *COL1A1, VTN, ITGA5, IL-4, TNF, ANGPT, VEGFA, and CTGF* genes:

In our previous study^11^, we used PAI scores (based on radiographic presentation) as one of the parameters to characterize patients into the healing and non-healing categories. PAI scores 1 and 2 were regarded as healing, whereas PAI scores 3 to 5 were considered non-healing. In the final step, we examine the correlation between PAI scores and relative baseline expression of *COL1A1, VTN, ITGA5, IL-4, TNF, ANGPT, VEGFA*, and *CTGF* genes. We found a strong, statistically significant (*p* < 0.05) positive correlation between *ITGA5* (0.96) expression and PAI score 3–5 (non-healing). None of the genes exhibited a statistically significant correlation with PAI scores 1–2.

## Discussion

This study was designed to see the association of the baseline expression profile of genes with periapical wound healing after surgical endodontic treatment. Analysis of baseline expression levels of the tested genes with healing status showed the mean relative expression of *VEGFA, ANGPT, TNF,* and *CTGF* was found to be significantly different (*p* < 0.05) between the healing group (72.99%) and the non-healing (94.42%) group. A statistically significant and strong positive correlation was also observed between *ITGA5* (0.96) expression and PAI score 3–5 in the non-healing group, while none of the genes exhibited a statistically significant correlation in the healing group.

**Figure 5:**
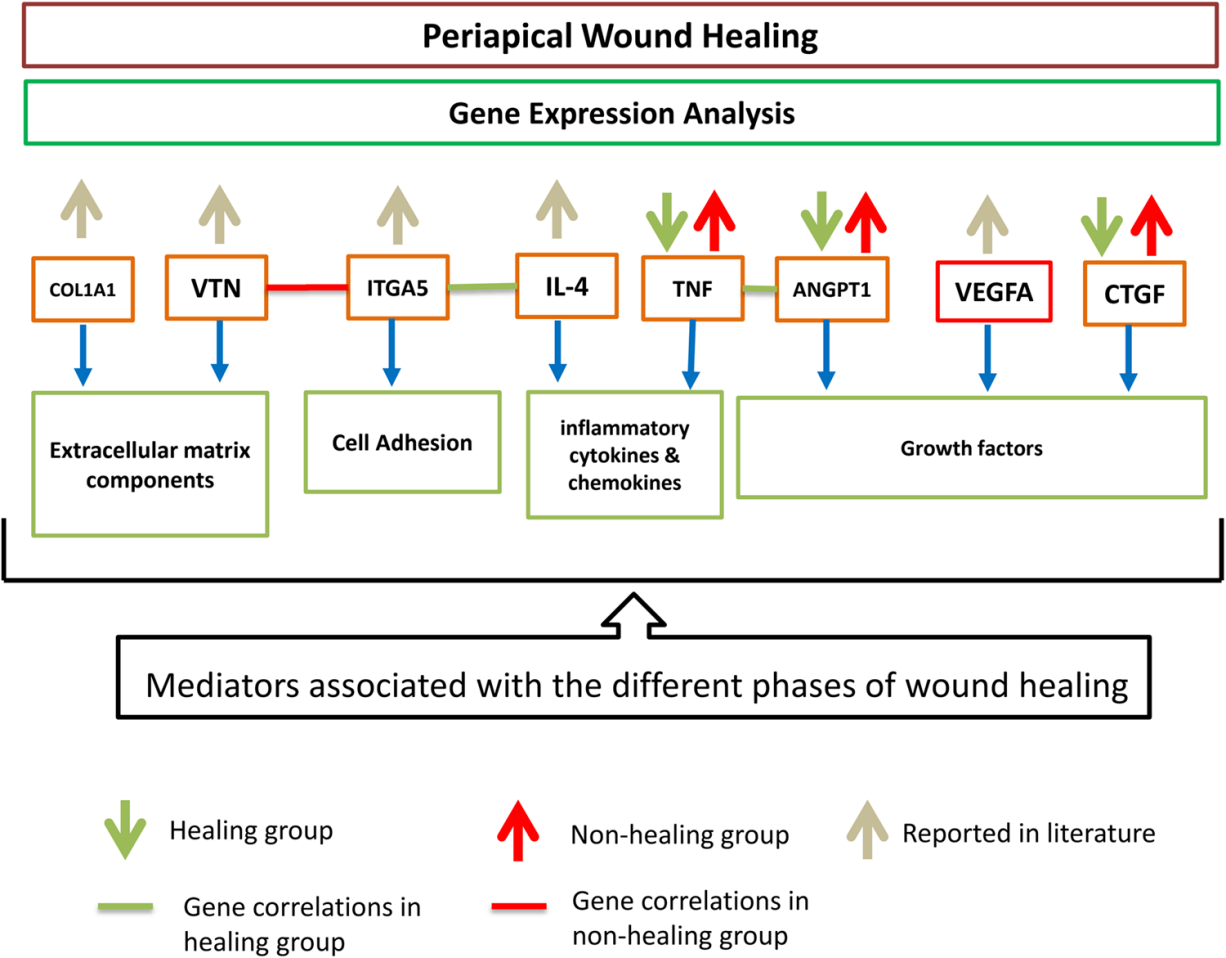
Graphical representation of the pathways of genes involved in periapical wound healing. It shows the baseline expression of wound healing marker genes viz. *COL1A1, VTN, ITGA5, IL-4, TNF, ANGPT1, VEGFA,* and *CTGF* in periapical granuloma after surgical endodontic treatment. In this study, we found the downregulation of *ANGPT1, TNF,* and *CTGF* genes in the healing group and upregulation of *ANGPT1, TNF,* and *CTGF* genes in the non-healing group. It also shows that periapical wound healing is associated with a decrease in the gene expression of the mediators of the inflammatory phase of wound healing (*TNF*), a decrease in mediators of angiogenesis (*ANGPT1*), and a decrease in the mediators of the remodeling phase of wound healing. Arrows directed upwards show the upregulation and arrows directed downwards show the downregulation of the gene expression on the analysis between the healing and non-healing groups. Green solid lines indicate that statistically significant positive correlations have been found in these pairs of genes in the healing group, whereas red solid lines indicate the statistically significant positive correlations association of a pair of genes in the non-healing group.

Wound healing after periapical surgery involves a complex interaction between cells and their surrounding microenvironment, patient response to periapical surgery follows a series of events involving chemotaxis of inflammatory cytokines, neutrophils, and growth factors to the periapical area followed by cellular differentiation resulting in the epithelial demarcation of the wound area. The final stage of wound repair and regeneration is achieved by vascular and functional matrix formation resulting in structural remodeling of the periapical tissues^15^. Structural remodeling of the periapical tissues is controlled by multiple genes involved in either up-regulation or down-regulation of stem cells to restore the form and function of the damaged periapical tissues^16,17^. The wound healing process involves a plethora of factors including the expression of the ECM, chemokines, cytokines, growth factors, remodeling enzymes, cellular adhesion molecules, and wound healing-associated genes such as *COL1A1, VTN*, *ITGA5, IL-4, TNF*, *ANGPT1, VEGFA*, and *CTGF*^18,19^. The genes *(COL1A1, VTN, ITGA5, IL-4, TNF, ANGPT1, VEGFA,* and *CTGF*) were selected for the baseline differential gene expression analysis for the healing and non-healing groups because they represented all major overlapping phases of wound healing processes, including inflammatory, proliferative, and remodeling phases^12,20^ (Fig. 5). It is important to note that these correlations were statistically significant correlations and may or may not have a biological basis. For instance, In the healing group, the positive correlation in the following genes could be explained on a biological basis since studies have shown that *IL-4* induces the expression of integrin 5 (*ITGA4*)^21^; and angiopoietin 1 could induce the *TNF*^22,23^ (Table 3). Similarly, in the non-healing group, the positive correlation between different genes can also be justified biologically. For instance, vitronectin (*VTN*) binds to integrin alpha 5 (*ITGA5*) that promotes cell adhesion in wound healing^24,25^.

In this study, the downregulation of *ANGPT1*,* TNF*, and *CTGF* genes in the healing group and upregulation of *ANGPT1*, *TNF*, and *CTGF* genes in the non-healing group were found. These findings show that periapical wound healing is associated with a decrease in the gene expression involved in the inflammatory phase of wound healing (*TNF*), angiogenesis (*ANGPT1*), or the remodeling phase of wound healing. In contrast, the upregulation of *ANGPT1*, *TNF*, and *CTGF* genes showed a statistically significant positive correlation with the non-healing group, hence, indicating that persistent activation of wound healing markers is associated with the inflammatory and remodeling phases leads to reduced healing in the periapical granuloma. Studies have reported the upregulation of genes, such as *COL1A1, VTN, ITGA5, IL-4,* and *VEGFA* to be associated with wound healing^26–28^, however, in this study, we did not find the difference in the expression of these genes in healing and non-healing groups (Fig. 5).

These gene expression patterns of the above-mentioned genes could be better understood by examining the pathophysiological roles of these genes of interest, for instance, Angiopoietin 1 is a growth factor encoded by *ANGPT1*, involved in the process of angiogenesis by controlling microvascular permeability. In this study, we found up-regulation of *ANGPT1* in the non-healing group, which may be associated with high vascularity as observed clinically in the periapical granuloma. This finding is in agreement with Al-Hassiny et al.^29^, who also demonstrated increased expression of *ANGPT1* in the inflamed dental pulp. The relative gene expression of other genes such as *TNF* and *CTGF* were also increased in the non-healing group. Being a multifunctional cytokine, *TNF* has been shown to have a role in the periapical bone resorption and stimulation of periapical granuloma^30^, while the connective tissue growth factor (*CTGF*) plays a role in the development of granulation tissue and angiogenesis^31^. These results are also supported by Garlet et al*.*^12^, who reported higher expression of *ITGA4, ITGA5, FGF7, TGFB1, TNF, CXCL11, COL1A1, COL5A1, VTN,* and *CTGF* genes in the periapical granulomas when compared with control samples. Furthermore, integrins are cell-surface proteins, which are involved in cell-to-cell cell-to-matrixtrix interactions, cellular signaling, and transportation. Integrins have alpha (α) and beta (β) subunits that are non-covalently linked to form αβ transmembrane units and mediate signaling events essential for stable cellular adhesion, spreading, migration, survival, proliferation, and differentiation. Integrin alpha-5 protein is encoded by the *ITGA5* gene. We found a strong positive correlation between *ITGA5* (0.96) and PAI scores 3–5 in the non-healing group. A similar association was demonstrated by Garlet et al.^12^, who found a fivefold or greater increase in the expression of *ITGA5* in periapical granuloma as compared to control samples.

There are certain limitations in this study that could be addressed in future research. First, surgical endodontic treatment was performed by conventional means, instead of the microsurgical approach owing to a lack of resources.

Secondly, the cone indicator was used to assess the healing on a digital radiograph and was used as a reference marker to ensure constant distance and angle between the x-ray cone and sensor on every shoot. Additionally, on all the recall images the tube current, voltage, and exposure time were the same. Still, since the obtained x-ray remains two-dimensional therefore there might be a high probability of missing some details which exist in the third dimension. Currently, for the evaluation of the periapical healing after surgical endodontic treatment and to reach the correct diagnosis, the cone beam computer tomograph is considered a standard of care. Numerous studies have shown that CBCT is significantly better in terms of sensitivity, diagnostic accuracy, and positive or negative predictive values than a digital periapical radiograph^32–34^. Barbat & Messer^35^ observed that it was difficult to detect even a large size periapical lesion in cancellous bone on X-ray until cortical bone became eroded. Similarly, Durack et al.^36^ compared the ability for periapical X-ray and CBCT with 180 or 360 rotations for the early detection of external inflammatory root resorption. They concluded that CBCT had higher positive and negative predictive values than periapical X-ray, besides any degree of CBCT rotation.

Third, the study was conducted on single-rooted upper/lower anterior teeth only, without considering multi-rooted posterior teeth. Fourth, the sample size of the study was small due to the unavailability of the patients, therefore, the results should be extrapolated carefully. Finally, the baseline expression profile of different genes was observed in the beginning, and later healing was assessed clinically and radiographically, further intervention for collecting tissue samples during the course of healing was not attempted because it could disturb healing and could be unethical to the patient.

## Conclusion

Overexpression of *ANGPT* and a strong positive correlation between *ITGA5* expression and PAI scores 3–5 in the non-healing group of patients suggest that early detection of this overexpression and correlation in a periapical granuloma tissue sample may indicate the prognosis of periapical wound healing after surgical endodontic treatment. Future studies with a large sample size are required to thoroughly evaluate periapical wound healing while keeping other micro-environmental factors in mind.

## Supplementary Information

Supplementary Information.

## Data Availability

All data is available in the supplementary files associated with the manuscript.
